# Supplementary figures and images for: Transcriptome sequencing and delimitation of sympatric Oscarella species (O. carmela and O. pearsei sp. nov) from California, USA
Source: PLoS One. 2017 Sep 11;12(9):e0183002. doi: 10.1371/journal.pone.0183002 (PMC5593202; doi:10.1371/journal.pone.0183002)

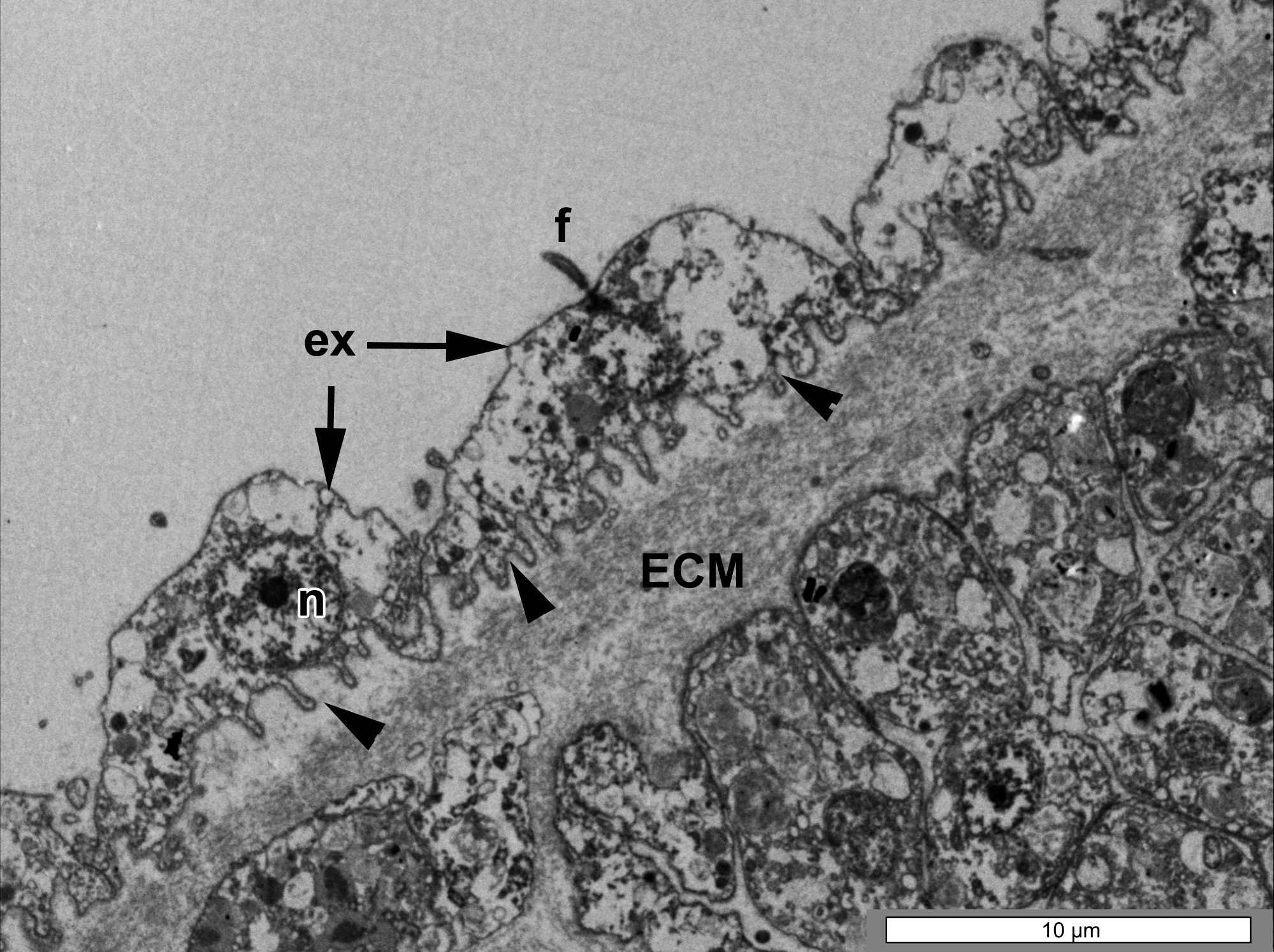

Supplement: S1 Fig — f–flagellum, n–nucleus. (TIF) [file pone.0183002.s001.tif]

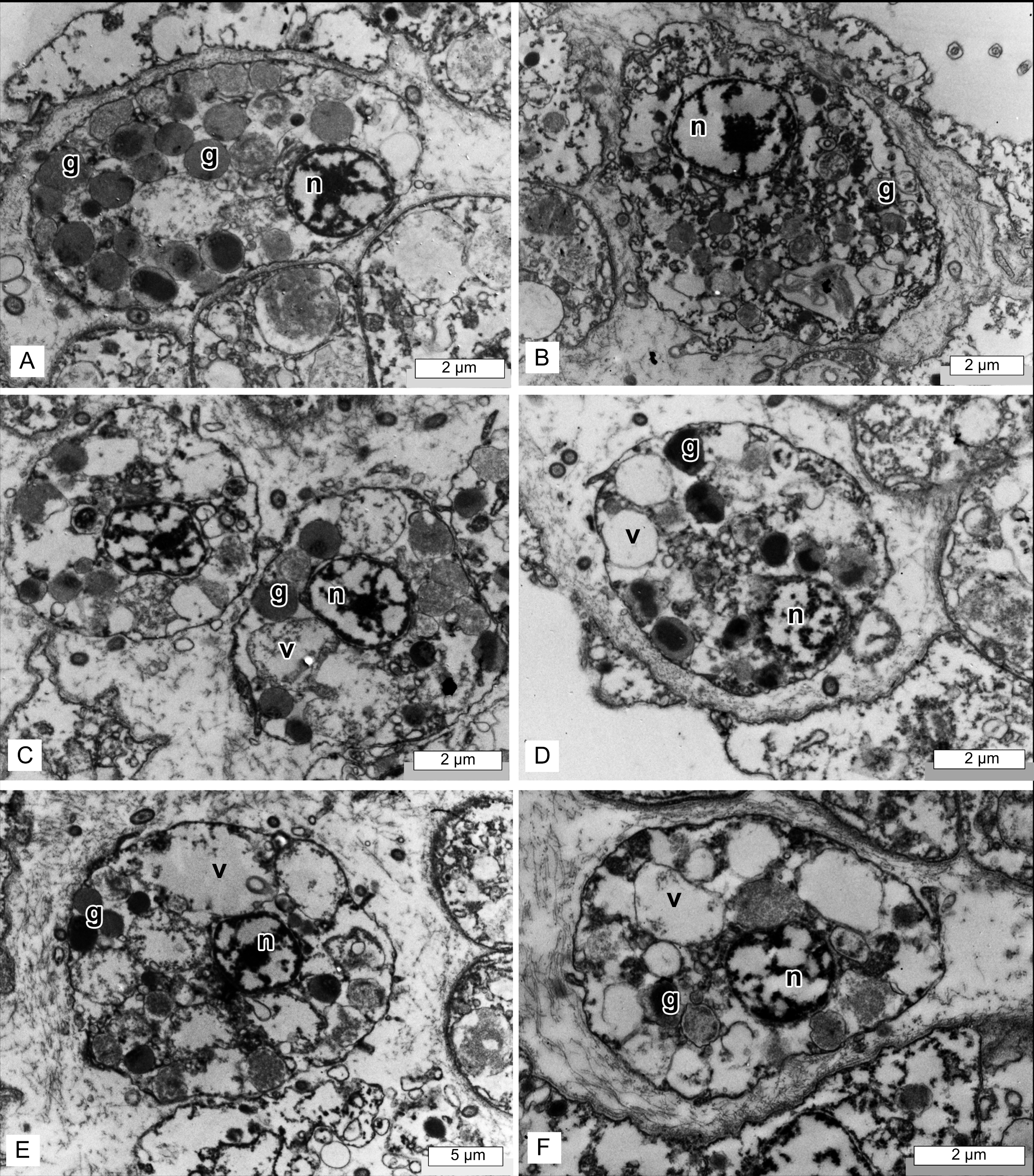

Supplement: S2 Fig — g–granule, n–nucleus, v–vacuole. (TIFF) [file pone.0183002.s002.tiff]

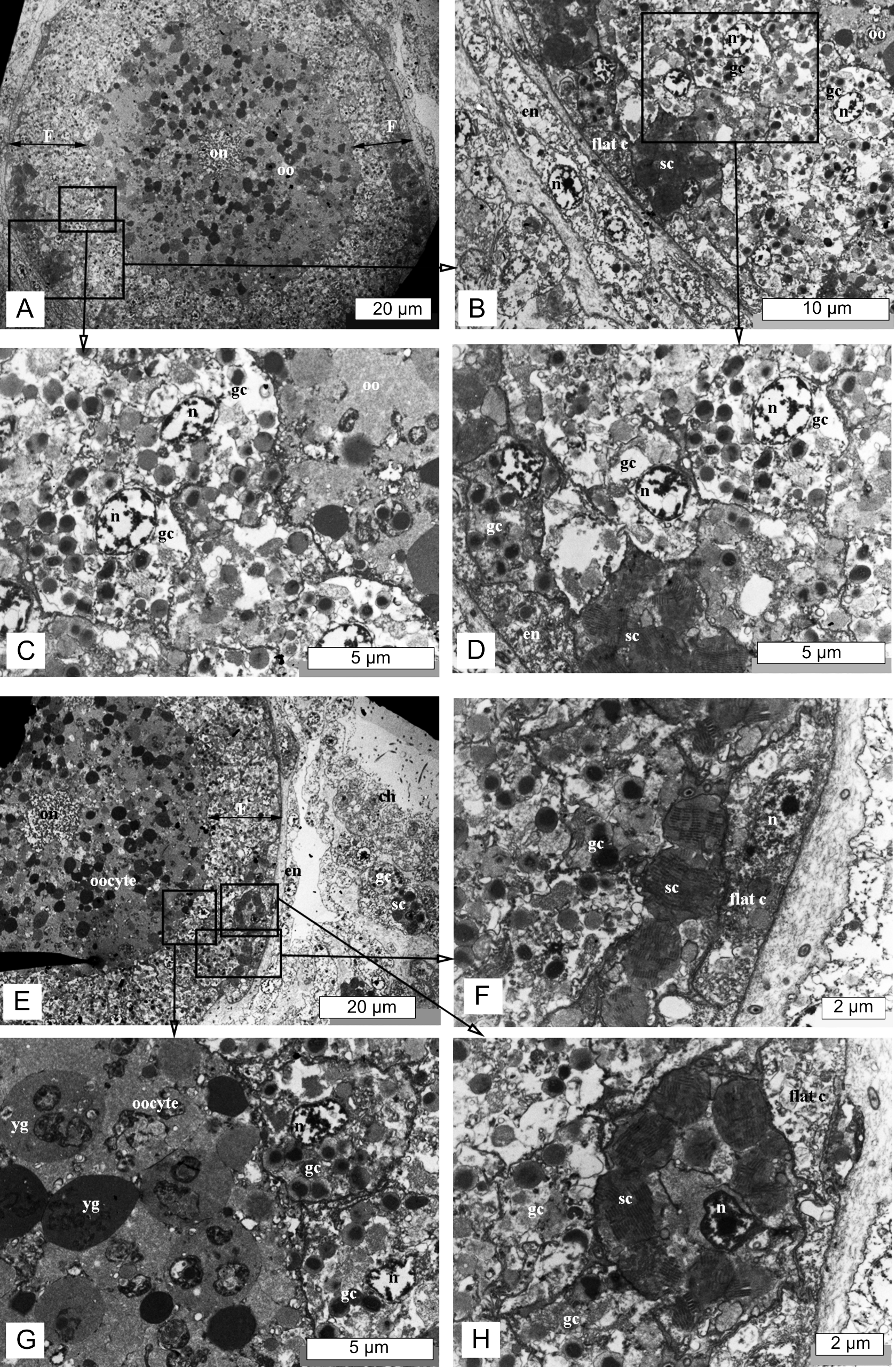

Supplement: S3 Fig — (A) Mature oocyte with two-layer follicle. (B)—(D) details of (A) showing granular and spherulous cells, composed internal layer of the follicle. (E) the part of a mature oocyte with follicle. (E)—(H) details of (E) showing granular and spherulous cells, composed internal layer of the follicle and external flat cells of a follicle (F), (H). ch–choanocytes, en–endopinacocytes, F–follicle, flat.c–flat cells of follicle, gc–granular cell, on–oocyte’s nucleus, oo–oocyte, n—nucleus, sc- spherulous cells with paracrystalline inclusions, yg–yolk granules. (TIFF) [file pone.0183002.s003.tiff]

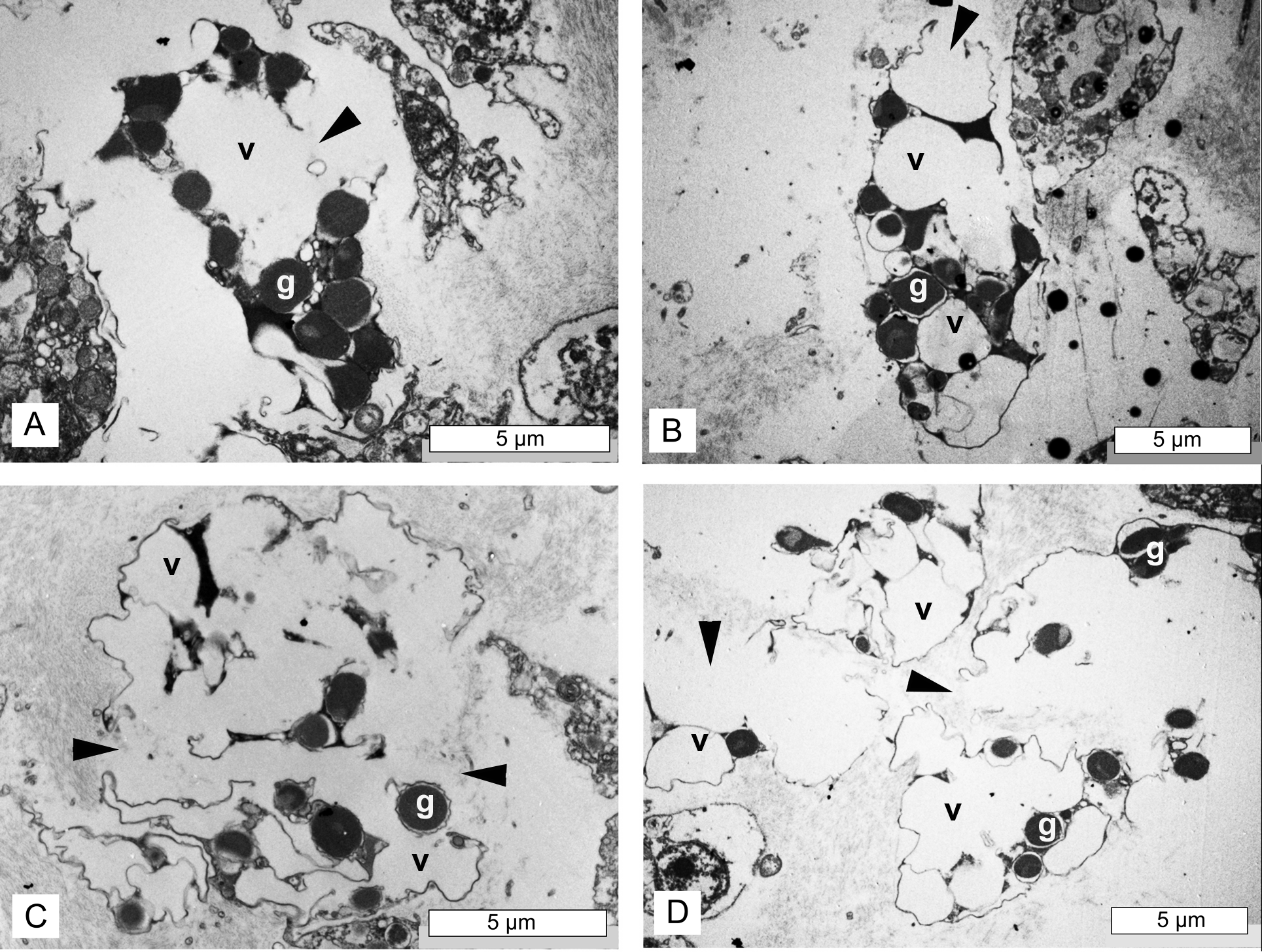

Supplement: S4 Fig — Arrowheads show the apparent release of vacuolar content in the mesohyl. g–granule, v–vacuole. (TIFF) [file pone.0183002.s004.tiff]
